# Novel Sylvatic Rabies Virus Variant in Endangered Golden Palm Civet, Sri Lanka

**DOI:** 10.3201/eid1712.110811

**Published:** 2011-12

**Authors:** Takashi Matsumoto, Kamruddin Ahmed, Omala Wimalaratne, Susilakanthi Nanayakkara, Devika Perera, Dushantha Karunanayake, Akira Nishizono

**Affiliations:** Oita University, Yufu, Japan (T. Matsumoto, K. Ahmed, A. Nishizono);; Medical Research Institute, Colombo, Sri Lanka (O. Wimalaratne, S. Nanayakkara, D. Perera, D. Karunanayake)

**Keywords:** zoonoses, viruses, rabies, *Paradoxurus zeylonensis*, palm civet, sylvatic, variant, Sri Lanka, molecular epidemiology

## Abstract

Information is scarce about sylvatic rabies virus in Asia and about rabies in palm civets. We report a novel sylvatic rabies virus variant detected in a golden palm civet in Sri Lanka. Evolutionary analysis suggests the virus diverged from canine rabies viruses in Sri Lanka in ≈1933 (range 1886–1963).

Rabies has been eliminated from domestic animals in industrialized countries, but sylvatic rabies remains an endemic disease. The ecology of rabies in wildlife populations and natural ecosystems is poorly understood (*1*), and, as a result, eliminating rabies from the wild is difficult. Little is known about sylvatic rabies in developing countries, where rabies takes its biggest toll on humans. Rabies is endemic to Sri Lanka and has been identified in different wild animals. However, all documented cases of rabies in wildlife in Sri Lanka have been considered a consequence of spillover from dogs. Rabies viruses circulating in this country are distinctly highly homogeneous (*2**,**3*).

Two species of palm civet are commonly found in Sri Lanka: the common palm civet, *Paradoxurus hermaphroditus*, which is widespread in southern Asia and Southeast Asia, and the golden palm civet, *P. zeylonensis*, which is indigenous to Sri Lanka. This species is closely related to the brown palm civet (*P. jerdoni*), which lives only in southern India (*4*). Moreover, 3 additional new species have been identified in Sri Lanka: the golden wet-zone palm civet (*P. aureus*), the golden dry-zone palm civet (*P. stenocephalus*), and the Sri Lankan brown palm civet (*P. montanus*) (*4*). Palm civets in Sri Lanka are, however, endangered because of hunting, parasitic diseases, and dwindling habitat. We report on a sylvatic rabies virus variant detected in a golden palm civet in Sri Lanka.

## The Study

On a November morning in 2009, a “wild cat” appeared in the garden of a basic health clinic in Moneragala district, Uva Province, Sri Lanka. The animal, which showed aggressive behavior, was suspected to be rabid and was thus killed to prevent transmission of rabies virus to humans. The animal’s head was packed in ice to avoid decomposition and sent to the Medical Research Institute (Colombo, Sri Lanka) for testing. We detected rabies virus in the animal’s brain by using the fluorescent antibody test and extracted viral RNA and DNA by using Trizol (Invitrogen, Carlsbad, CA, USA). The rabies virus from this sample was designated as H-1413-09. The whole genome of the virus was sequenced directly from the sample as described (*5*).

To confirm the species of the rabid animal, we determined the nucleotide sequence of the mitochondrial cytochrome *b* (*cytb*) gene and performed a BLAST search (www.ncbi.nlm.nih.gov/blast/Blast.cgi) for similarity with other sequences. By aligning nucleotide sequences of the *cytb* gene of mitochondrial DNA of domestic cat, jungle cat, fishing cat, Asiatic golden cat, marbled pole cat, European pole cat, lynx, puma, leopard, African lion, tiger, jaguar, civet, and palm civet with ClustalW2 (www.ebi.ac.uk/clustalw), we designed primer Felis *cytb*-F, 5′-ATGACCAACATTCGAAAATCACACC-3′ (nt 1–25), and primer Felis *cytb*-R, 5′-CAATAATGCCTGAGATGGGTATTAG-3′ (nt 1093–1,117). Using these primers, we performed PCR as follows: initial denaturation at 94°C for 2 min, followed by 94°C for 30 s, 55°C for 30 s, and 72°C for 1 min for 35 cycles, followed by a final extension at 72°C for 5 min. PCR generated a 1,117-bp fragment from which a 1,004-nt sequence was determined. Analysis showed that the sequence has 100% identity with the partial (224-nt) sequence of the *cytb* gene of *P. zeylonensis* (GenBank accession no. FJ881681); this is the only sequence available for *P. zeylonensis*. The sequence also has 95% identity with *P. jerdoni* and 90%–92% identity with *P. hermaphroditus*.

We performed an evolutionary analysis by using the N gene. We inferred a maximum clade credibility phylogenetic tree by using the Bayesian Markov chain Monte Carlo method available in BEAST version 1.6.1 (*6*). The analysis used a relaxed (uncorrelated lognormal) molecular clock and GTR + Γ + I model of nucleotide substitution. We selected the model on the basis of Akaike Information Criterion by using jModelTest software (*7*). All chains were run for 9 × 10^7^ generations and sampled every 3,000 steps. This procedure resulted in an effective sample size of >2,000 for all estimated parameters. The posterior densities were calculated with 10% burn-in and checked for convergence by using Tracer version 1.5 (http://beast.bio.ed.ac.uk/Main_Page). The mean rate of nucleotide substitution estimated for the N gene was 2.2 × 10^4^ substitutions/site/year (95% highest posterior density [HPD] values 1.3–3.2 × 10^4^ substitutions/site/year). This rate is in agreement with previous findings (*8*). Approximately 155.5 years ago (95% HPD 91.3–249.5 years)—that is, circa 1854 (95% HPD range 1760–1918)—rabies viruses from Sri Lanka and southern India diverged from their most recent common ancestor. Approximately 76.4 years ago (95% HPD 46.4–123.0 years)—that is, in ≈1933 (95% HPD range 1886–1963)—strain H-1413-09 diverged from canine rabies virus in Sri Lanka.

Compared with the genome sequence of rabies virus strain H-08-1320 from Sri Lanka, the genome sequence of strain H-1413-09 had a nucleotide deletion at residue 2,417 and an addition at nt 11,807. As a result, the start and stop signals of the mRNA and the start and stop positions of coding sequences of different genes were altered (Table 1). These altered start and stop codons advanced the coordinates of the intergenic signal of relevant intergenic regions. The P-M region and terminal sequence of strain H-08-1320 were 1 nt shorter (91 nt) and longer (136 nt), respectively. The nucleotide and amino acid identities between the coding regions of strains H-1413-09 and H-08-1320 are shown in Table 1. The substitutions detected in the deduced amino acid sequences of H-1413-09 were compared with the genomic sequence of H-08-1320 (Table 2). By using the MEGA5 Tamura-Nei model (www.megasoftware.net), we determined the genetic distance of the N gene to determine whether strain H-1413-09 is more diverse than other rabies viruses in Sri Lanka (Figure). The rate of variation among sites was modeled with a gamma distribution (shape parameter = 0.5). The genetic distances between strain H-1413-09 and other rabies viruses from Sri Lanka (0.027–0.036) were greater than the genetic distances among other rabies viruses (0.001–0.011). These results support our finding that strain H-1413-09 differs from other rabies viruses circulating in Sri Lanka.

**Table 1:** Percentage identity shared between genes of 2 rabies virus strains from Sri Lanka, H-08-1320 and H-1413-09, by gene coding region*

Gene	H-1413-09			H-08-1320			% Identity	
	Coding region	Start codon, stop codon		Coding region	Start codon, stop codon		Nucleotide	Amino acid
N	71–1,423	ATG, TGA		71–1,423	ATG, TGA		99.1	97.1
P	1,514–2,404	ATG, TGA		1,514–2,404	ATG, TGA		99.1	97.5
M	2,496–3,104	ATG, TAA		2,497–3,105	ATG, TAA		98.0	96.9
G	3,317–4,891	ATG, TGA		3,318–4,892	ATG, TGA		98.7	97.4
L	5,407–11,793	ATG, TGA		5,408–11,794	ATG, TAA		99.0	97.0

*H-08-1320 is a human strain typical of canine rabies virus circulating in Sri Lanka; H-1413-09 is a novel sylvatic rabies virus variant from a golden palm civet in Sri Lanka.

**Table 2:** Substitutions in genome sequence of rabies virus strain H-1413-09 from Sri Lanka, compared with genome sequence of strain H-08-1320*

Protein, amino acid substitution	Site/domain/region of protein†
N	
Leu_80_ → Phe_80_	
Glu_110_ → Asp_110_	
Ile_246_ → Val_246_	
Ala_372_ → Val_372_	Antigenic site I
P	
Gln_167_ → Arg_167_	N protein binding site in variable domain II
M	
Ile_16_ → Ala_16_	
Pro_19_ → Ser_19_	
Ile_55_ → Val_55_	
Lys_77_ → Arg_77_	
G	
Val_193_ → Ile_193_	
Arg_264_ → His_264_	
Ile_449_ → Thr_449_	Transmembrane region
Thr_459_ → Ile_459_	Transmembrane region
Ala_467_ → Thr_467_	Cytoplasmic domain
Glu_475_ → Gly_457_	Cytoplasmic domain
Asn_499_ → Ser_499_	Cytoplasmic domain
L	
Ser_26_ → Pro_26_	
Ile_49_ → Leu_49_	
Cys_137_ → Tyr_137_	
Leu_222_ → Ile_222_	
Ser_312_ → Gln_312_	Conserved domain I
Glu_313_ → Lys_313_	Conserved domain I
Ser_314_ → Ala_314_	Conserved domain I
Arg_315_ → Glu_315_	Conserved domain I
Val_317_ → Phe_317_	Conserved domain I
Lys_1056_ → Arg_1056_	Conserved domain IV
Thr_1137_ → Val_1137_	Conserved domain V
Ala_1520_ → Glu_1520_	
Ile_1555_ → Val_1555_	
Leu_1570_ → Met_1570_	
Met_1577_ → Leu_1577_	
Lys_1625_ → Arg_1625_	
Asn_1763_ → Asp_1763_	
Arg_1876_ → His_1876_	
Asn_2023_ → Asp_1763_	
Gly_2098_ → Arg_2098_	
Leu_2113_ → Phe_2113_	

*H-08-1320 is a human strain typical of canine rabies virus circulating in Sri Lanka; H-1413-09 is a novel sylvatic rabies virus variant from a golden palm civet in Sri Lanka. †Blank spaces indicate no site/domain/region has been identified in that portion of the protein.

**Figure:**
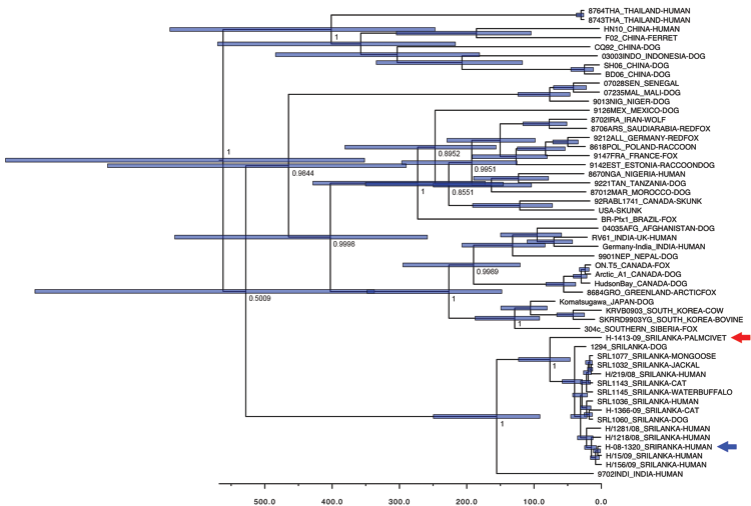
Bayesian maximum-credibility tree representing the genealogy of rabies virus as obtained by analyzing nucleotide sequences of full N gene sequences (1,350 nt). Nodes correspond to mean age at which lineages are separated from the most recent common ancestor; blue horizontal bars at nodes represent 95% highest posterior density of the most recent common ancestor. Numbers at main nodes represent posterior values. Horizontal axis at bottom represents time scales in years, beginning at 2010. Red arrow indicates strain H-1413-09; blue arrow indicates strain H-08-1320. Nucleotide sequence data for strains from Sri Lanka appear in nucleotide sequence databases of DNA DataBank of Japan, European Molecular Biology Laboratory, and GenBank with accession no. AB635373 (rabies virus strain H-1413-09), AB638767 (strain H-219-08), AB638768 (strain H-1218-08), AB638769 (strain H-1281-08), AB638770 (strain H-15-09), AB638771 (strain H-156–09), AB638772 (strain H-1366-09), and AB636165 (golden palm civet [*Paradoxurus zeylonensis*] strain H-1413-09).

## Conclusions

Rabies virus probably survives favorably in the wild because it can infect a large spectrum of animals, thereby maximizing replication and dispersal opportunities (*9*). Most viruses replicate poorly when transferred to new hosts, but greater genetic variation assists in such species adaptation (*10*). Increased mutation in an RNA virus like rabies virus can give rise to variants with altered levels of fitness to persist and spread. A large number of substitutions were found in strain H-1413-09 compared with strain H-08-1320; these substitutions might represent changes that resulted from species adaptation. Phylogenetic analysis and comparative sequence data indicated that strain H-1413-09 is a variant rabies virus.

Palm civets are facing extinction in Sri Lanka because the species is losing its habitat, being hunted for its meat, and dying of parasitic diseases (www.sundaytimes.lk/090118/Plus/sundaytimesplus_01.html). Our study indicates that rabies might be another risk factor for extinction of these animals. Identification of a variant rabies virus in wildlife has serious implications for rabies control in Sri Lanka. Identification of such a virus would help provide epidemiologic data about the spread of rabies and its incursion into new geographic regions and would justify allocation of increased resources to help control rabies (*11**,**12*).

Several rabies virus variants associated with wildlife are known in the Americas and Africa (*1**,**13**–**15*), and this report identified classical sylvatic rabies in Asia. Whether *P. zeylonensis* is a reservoir of rabies virus or represents spillover from another animal deserves extensive investigation. The detection of rabies in wildlife indicates that much remains to be discovered in the tropical ecosystem of Sri Lanka. The circulation of a sylvatic variant rabies virus may be another hurdle in the rabies-control effort in Sri Lanka.
